# Asymmetric TMO–Metal–TMO Structure for Enhanced Efficiency and Long-Term Stability of Si-Based Heterojunction Solar Cells

**DOI:** 10.3390/ma16165550

**Published:** 2023-08-09

**Authors:** Yoon-Chae Jung, Young-Jin Yu, Yu-Kyung Kim, Jin Hee Lee, Jung Hwa Seo, Jea-Young Choi

**Affiliations:** 1 Department of Metallurgical Engineering, Dong-A University, Busan 49315, Republic of Korea; 2 Department of Chemical Engineering (BK21 Four Graduate Program), Dong-A University, Busan 49315, Republic of Korea; 3 Department of Physics, University of Seoul, Seoul 02504, Republic of Korea; 4 Department of Materials Sciences & Engineering, Dong-A University, Busan 49315, Republic of Korea

**Keywords:** transition metal oxide, asymmetric, passivation, long-term stability, silicon, heterojunction solar cell

## Abstract

In this study, we fabricated Si-based heterojunction solar cells (HSCs) with an asymmetric TMO–metal–TMO (TMT) structure using both MoO_3_ and V_2_O_5_ as the hole-selective contacts. Our HSCs offer enhanced long-term stability and effective passivation for crystal defects on the Si sur-face. We analyzed the oxygen vacancy state and surface morphology of the MoO_3_- and V_2_O_5_-TMO thin films using X-ray photoelectron spectroscopy and atomic force microscopy to investigate their passivation characteristics for Si surface defects. From the measured minority carrier lifetime, V_2_O_5_ revealed a highly improved lifetime (590 μs) compared to that of MoO_3_ (122.3 μs). In addition, we evaluated the long-term stability of each TMO thin film to improve the operation stability of the HSCs. We deposited different types of TMOs as the top- and bottom-TMO layers and assessed the effect of the thickness of each TMO layer. The fabricated asymmetric TMT/Si HSCs showed noticeable improvements in efficiency (7.57%) compared to 6.29% for the conventional symmetric structure which used the same TMO material for both the top and bottom layers. Furthermore, in terms of long-term stability, the asymmetric TMT/Si HSCs demonstrated an efficiency that was 250% higher than that of symmetric TMT/Si HSCs, as determined via power conversion efficiency degradation over 2000 h which is mainly attributed by the lower oxygen vacancy of the top-TMO, V_2_O_5_. These results suggest that the asymmetric TMT structure is a promising approach for the fabrication of low-cost and high-efficiency Si-based HSCs with enhanced long-term stability.

## 1. Introduction

In recent decades, crystalline silicon (c-Si) has become a prominent technology in the global photovoltaic market due to its numerous advantages, including high efficiency, long-term stability, abundant and non-toxic silicon material, and a favorable energy bandgap that allows for effective light absorption in the visible region [1]. c-Si solar cells can be classified into homojunction and heterojunction solar cells based on the type of junction material. The p–n junction-based homojunction c-Si solar cell is favored for its high power conversion efficiency (PCE) and stability [2]. However, the production of homojunction solar cells is costly, as it requires a complex manufacturing process that inevitably includes high-temperature doping processes, ≥800 °C [1,3]. To address the limitations of conventional homojunction c-Si solar cells, researchers have been exploring the use of heterojunction structures to reduce manufacturing costs and improve PCE. Generally, heterojunctions can be fabricated through a simpler and less costly process, and materials that allow for the formation of junctions at relatively low temperatures are attracting significant attention. These materials include graphene [4], transparent conductive oxides (TCOs) [5,6], transition metal oxides (TMOs) [7,8], organic materials [9,10], and perovskite [11,12]. Among the materials mentioned, transition metal oxides (TMOs) have been shown to be effective carrier-selective materials that provide electrical properties and photovoltaic performance that are suitable for dopant-free Si-based heterojunction solar cells (HSCs) [13,14,15]. TMOs have a wide range of work functions, from 3 to 7 eV, which allows for the formation of both hole- and electron-selective contacts by selecting appropriate materials [16]. TMOs with high work functions close to 7 eV, such as molybdenum trioxide (MoO_3_), vanadium pentoxide (V_2_O_5_), and tungsten trioxide (WO_3_), are particularly suitable for hole-selective layers [17]. Additionally, the relatively large energy bandgap of TMOs (E_g_ > 3 eV) also minimizes parasitic absorption when deposited as front contacts in HSCs [16,18]. Moreover, the presence of TMOs on Si surfaces leads to the formation of a thermodynamically spontaneous silicon oxide (SiO_x_, where 0 ≤ x ≤ 2) layer at the TMO/Si interface, which contributes as a passivation layer for the Si surface [13,19]. TMOs provide two types of passivation features for the Si surface: (1) chemical passivation, which decreases the density of Si surface defects through chemical bonding between the oxygen in TMOs and the dangling bonds on the Si surface; and (2) field-effect passivation, which arises from the high work function of the TMO and produces an imbalance of electron and hole carrier concentrations at the TMO/Si interface [20,21]. These properties of TMOs as passivation layers and as carrier-selective contacts can reduce carrier recombination on the Si surface, increase the efficiency of charge carrier extraction, and thus enhance the PCE of HSCs [22,23].

Despite the advantages of TMO introduced HSCs, the development of high-efficiency TMO/Si HSCs faces several critical challenges originated from TMO layers, including the following: (1) poor passivation effect (i.e., high carrier recombination velocity); (2) limited long-term stability; and (3) high sheet resistance (R_sheet_). First, the low passivation effect results in a reduced PCE because it decreases the minority carrier lifetime [15]. The formation of a low-quality SiO_x_ passivation layer during the deposition of TMOs on the Si surface is the root cause of this low passivation effect [24]. Second, the long-term stability of the solar cells is limited by the high oxygen vacancy density in TMOs formed during deposition by thermal evaporation, which reacts with airborne molecules (e.g., water) over time, degrading the TMO work function and ultimately the stability of the HSC [25]. Lastly, as a dielectric material, TMO thin film (TF) inherently exhibits a high R_sheet_ which inevitably hinders the extraction of photogenerated carriers from inside Si [26]. To achieve high-efficiency Si-based HSCs, it is crucial to develop methods that improve (1) the passivation performance for the Si surface; (2) the efficiency of charge carrier extraction; and (3) the long-term stability of the fabricated HSCs. The aim of this study is to simultaneously achieve improved efficiency and long-term stability of TMO/Si HSCs after introducing multilayered TMO with two different TMOs, namely, vanadium pentoxide (V_2_O_5_, VO) and molybdenum trioxide (MoO_3_, MO). The optimal passivation characteristics of TMOs were attained through morphological and stoichiometric analysis of the two deposited TMO TFs (VO and MO), which were produced at low temperatures below 125 °C. However, it was believed that the single-layer TMO structure would limit the PCE of the HSC. Therefore, a TMO–Metal–TMO (TMT)-structured HSC was fabricated to further improve PCE. The TMT/Si HSC was fabricated by introducing an asymmetric TMT structure through thermal deposition. MO was used as the bottom-TMO (BTMO) due to its relatively low R_sheet_, while VO was used as the top-TMO (TTMO) to enhance field-effect passivation. We confirmed that the VO TF used as a TTMO layer also offered enhanced long-term stability due to its low oxygen vacancy density. From the report, we successfully demonstrate that the introduction of an asymmetric TMT structure in a Si-based HSC significantly improves the PCE and long-term stability of the HSC, even with simple and low-temperature fabrication processes.

## 2. Experimental Section

### 2.1. Material and Sample Preparation

For Si, a double-sided polished n-type CZ Si wafer with a (100) orientation, thickness of 280 μm, and resistivity of 1.7–2.3 Ω·cm was employed. For sample preparation, the Si wafer was cut to the size of 2×2 cm for HSC fabrication, and it was cleaned through ultrasonication for 15 min with acetone, methanol, and distilled water (DI water) in the order. The wafer then underwent a standard RCA cleaning process ($NH_{4}OH:H_{2}O_{2}:DI\;water=1:1:5$) for 15 min to remove organic residues on the wafer, and the wafer was dipped for 1 min in 1% diluted HF solution to remove the native oxide. After the HF dip process, the VO and MO (Alfa Aesar, Haverhill, MA, USA, 99.995% powder) TFs were deposited using a molybdenum (Mo) boat via vacuum thermal evaporation. Each TMO was deposited at a rate of 0.2 Å/s and a vacuum level of $1\times 10^{-6}\;\mathrm{mbar}$ at the target heating temperatures (no heating, 75 °C, and 125 °C). In the case of the metal electrode, Al and Ag were deposited to a thickness of 200 nm. at a rate of 1.0 Å/s and a vacuum level of $1\times 10^{-6}\;\mathrm{mbar}$. To examine the change in passivation characteristics with Si substrate temperature, HSC with a TMO/Si/TMO sandwich structure was fabricated by deposition after heating the substrate up to different target temperatures such as room temperature (RT, i.e., no-heating), 75, and 125 °C.

### 2.2. Characterization

The minority carrier lifetimes ($\tau_{eff}$) of the Si samples with a single-TMO TF and a TMT multilayer were measured with a photoconductance decay system (WCT-120, Sinton Instrument Inc., Boulder, CO, USA). The surface morphology of the TMO TF was analyzed using atomic force microscopy (AFM, tapping mode, Multi-Mode V, Veeco, Oyster Bay, NY, USA). Furthermore, the chemical composition and oxygen deficiency of TMO TFs were analyzed using X-ray photoelectron spectroscopy (XPS, ESCALB-250XI, Thermo-Fischer Scientific, Waltham, MA, USA). The sheet resistance (R_sheet_) of the TMO TF was measured using the transmission line method (TLM) after setting the voltage scan range −1.0 to 1.5 V. Finally, the PCE of the fabricated HSCs was investigated using a solar simulator under an air mass (AM) 1.5 G condition. (Note: to obtain the reliability of data, 3 to 5 identical samples were fabricated for each characterization.)

## 3. Results and Discussion

### 3.1. Effect of TMO on the Performance of TMO/Si HSCs

**Figure 1:**
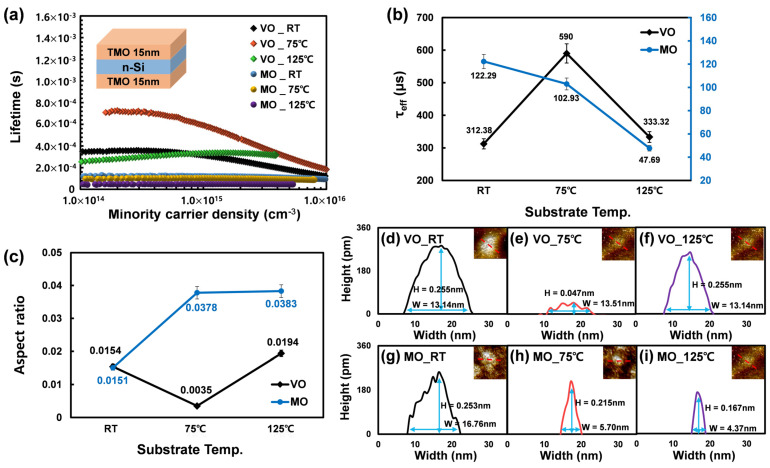
(**a**) $\tau_{eff}$ of two TMOs (VO and MO) deposited at different temperatures (RT, 75, and 125 °C); (**b**) extracted $\tau_{eff}$ values at the carrier injection level of 1.5 × 10^15^ cm^−3^; (**c**) aspect ratio of TMO islands; (**d**–**i**) the width and height of each TMO island deposited at various substrate temperatures.

In this study, to evaluatie the Si surface passivation characteristics of two different TMOs according to the deposition temperatures, sandwich structured samples were fabricated with MO or VO deposited on both sides of the Si substrate at substrate temperatures of RT, 75, and 125 °C. The minority carrier lifetimes ($\tau_{eff}$) of the samples were measured, and the results are presented in Figure 1a,b. In Figure 1b, for MO, the highest $\tau_{eff}$ (122.3 μs) was measured at RT, followed by a decrease in $\tau_{eff}$ with increasing substrate temperature. However, for VO, the highest $\tau_{eff}$ (590 μs) was measured at 75 °C. This substrate temperature-dependent $\tau_{eff}$ behavior is attributed to the change in the initial TMO/Si interface area (i.e., active region) for supplying oxygen atoms to the Si substrate as the aspect ratio (AR) of the initially deposited TMO nano island changes with increasing substrate temperature [24]. Figure 1d–i shows the AFM images of the nano islands according to the substrate temperature. Figure 1c reveals that the AR of the nano island is lowest at 75 °C and RT in the case of VO and MO, respectively, which is consistent with the $\tau_{eff}$ result shown in Figure 1b. Furthermore, in the case of VO, the lowest AR was observed at 75 °C, which confirms that the initially depositing VO can supply oxygen atoms to the Si surface more efficiently than the equivalent case in MO.

**Figure 2:**
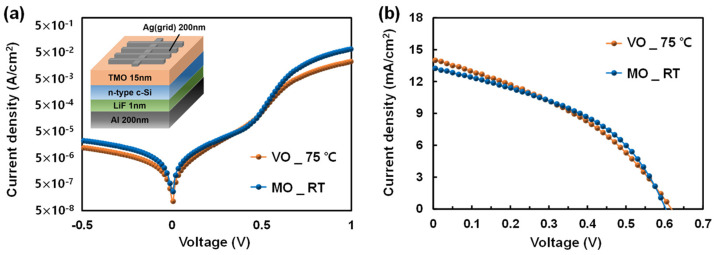
Current density–voltage (J–V) curves of TMO/Si HSCs under (**a**) dark and (**b**) light (AM1.5) conditions.

However, despite the low AR of the VO nano island, the Gibbs formation energy (ΔG) for the formation of SiO_2_ from MO ($\Delta G^{MO\to SiO_{2}}=-406\;\mathrm{kJ}/\mathrm{mol}$) is more negative than that from VO ($\Delta G^{VO\to SiO_{2}}=-285\;\mathrm{kJ}/\mathrm{mol}$) [21]. Therefore, even though the formation of SiO_2_ from MO is a thermodynamically more favorable, it is believed that the enhanced passivation performance of VO is due to factors other than SiO_x_ formation—specifically, the improved field-effect passivation of VO. The characteristics of the enhanced field-effect passivation of VO will be further discussed in Section 3.2. The performances of VO/Si and MO/Si HSCs were evaluated by depositing VO and MO on the Si surface, respectively, to analyze the relationship between the passivation characteristics of the TMO TFs and the PCE of the fabricated HSCs. Figure 2 shows the current density-voltage (J–V) curves, while Table 1 provides details on the solar cell performance parameters of the TMO/Si HSCs under both dark and illuminated conditions. Despite the exceptional Si surface passivation performance of VO as demonstrated in Figure 1b, the actual PCE of the MO/Si HSC was slightly higher than that of the VO/Si HSC, as shown in Table 1.

**Figure 3:**
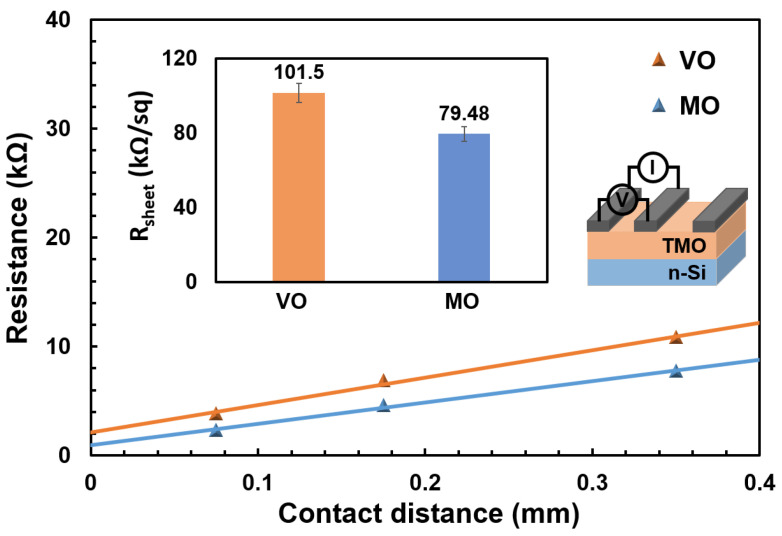
Sheet resistance of TMO TFs extracted from the ohmic current–voltage responses from TLM measurements (inset: structure of the fabricated TLM sample).

The performance parameters of the solar cells listed in Table 1 indicate that despite the better passivation performance of VO, it does not translate into an improvement in the actual PCE of the VO/Si HSC, which can most probably be attributed to the high series resistance (R_s_) of the VO/Si HSC. This demonstrates that the VO material itself has a high parasitic resistance in the device compared to MO. The R_sheet_ of each TMO TF was determined through transmission line method (TLM) measurements, as shown in Figure 3 [27]. By comparing the R_sheet_ of each TMO TF, it was found that VO has a R_sheet_ that is 20% higher than that of MO followed by relatively lower charge extraction efficiency with VO TF. The high R_sheet_ of the VO TF is caused by its lower density of oxygen vacancies compared to that of the MO. To further examine these relationships, X-ray photoelectron spectroscopy (XPS) spectra were analyzed for each TMO TF.

**Figure 4:**
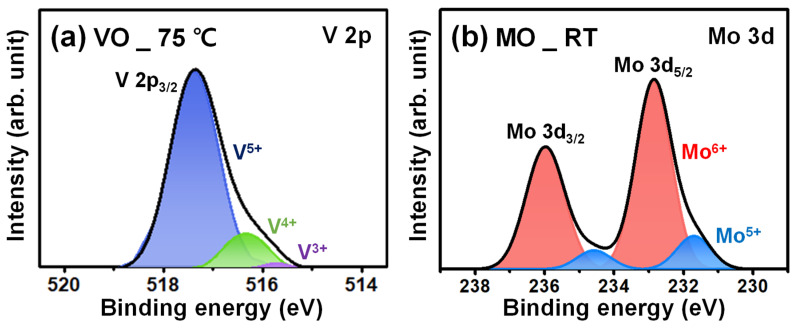
(**a**) V(2p) XPS spectra of VO TF deposited at a substrate temperature of 75 °C and (**b**) Mo(3d) XPS spectra of MO TF deposited at RT.

**Figure 5:**
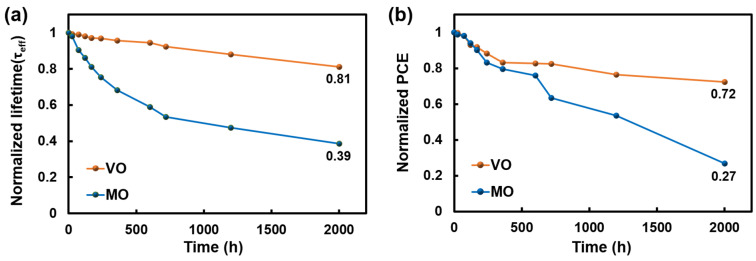
(**a**) Degradation of $\tau_{eff}$ and (**b**) PCE degradation of TMO/Si HSCs over 2000 h.

The XPS spectra of V 2p and Mo 3d are shown in Figure 4, and the integrated peak area ratio of the oxidation states of the two TMOs is presented in Table 2. In the case of VO, the V^5+^ ratio, which forms complete stoichiometric V_2_O_5_ in the deposited VO, was as high as 88.3%. However, for MO, the Mo^6+^ ratio, which forms MoO_3_, was 80.5%, which is lower than the oxidation state ratio of VO. The difference in these values indicates that more metallic TFs were formed with MO compared to VO, which is believed to be the reason for the higher R_sheet_ of VO TF. The comparison of the resistance and passivation characteristics of the respective TMO TF, as well as the PCE of TMO/Si HSCs, confirm that the implementation of low resistance TMO is critical for high-efficiency TMO/Si HSCs. However, in terms of the TMO/Si HSC efficiency, considering the low PCE value of 3.46% despite the comparatively low R_s_, further improvement in PCE should be required. In addition, the high density of oxygen vacancy (i.e., Mo^5+^) in TMO, which results in low R_sheet_ in MO TFs, degrades their long-term stability through a reaction with airborne molecules such as water when exposed to the ambient environment, thereby lowering the PCE of the fabricated HSC over time [25]. Figure 5a depicts the change in $\tau_{eff}$ over time for Si samples deposited with VO or MO for both sides, respectively, and as shown in the graph, for MO, $\tau_{eff}$ is measured at 39% of the initial level over the 2000 h measurement. In contrast, in the case of VO with a relatively low oxygen vacancy density, $\tau_{eff}$ is maintained at a higher level, which is 81% of the initial level, and comparison of these two results clearly indicate poor stability of MO. The degradation of the TMO over time is more pronounced from the change in HSC efficiency over time, as shown in Figure 5b. After 2000 h, PCE of the MO/Si HSC was measured at 27% of the initial level, whereas that of VO/Si HSC was at 72%. This suggests that excessive formation of high oxygen vacancies in MO considerably degrades the passivation performance and PCE. Thus, for fabrication of high-efficiency TMO/Si HSCs with long-term stability, techniques to simultaneously achieve (1) low TMO resistance; (2) improved Si surface passivation; and (3) low oxygen vacancy density must be developed.

### 3.2. Effect of TMO Thickness on TMT/Si HSC Performance

**Figure 6:**
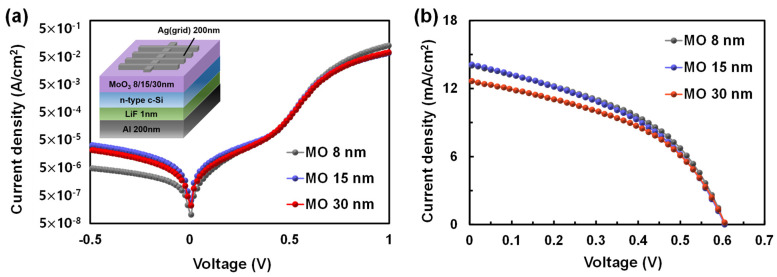
J–V curves of the MO/Si HSCs according to MO layer thickness under (**a**) dark and (**b**) light (AM1.5) conditions.

As discussed in Section 3.1, the VO TF offers excellent Si surface passivation characteristics, but a high resistance value; in contrast, the MO TF provides a low resistance, but exhibits poor Si surface passivation performance. In this study, we endeavored to build an asymmetric TMT-structured HSC that uses both TMOs (MO and VO) to increase device performance and long-term stability by exploiting the properties of the two TMOs. In the design of the HSC structure, we aimed to further improve the charge carrier extraction efficiency of the fabricated HSC by sandwiching the Ag metal layer as a conductive layer between the two TMO TFs [28,29]. For the fabrication of the asymmetric TMT/Si HSC, a low R_sheet_ MO TF was applied as the BTMO, and a VO TF with a low oxygen vacancy density was deposited as the TTMO to overcome the insufficient Si surface passivation of the bottom MO TF through the enhanced field-effect passivation. The thickness of each TMO TF was optimized to fabricate an asymmetric TMT/Si HSC; to this end, MO/Si HSCs were first fabricated, and the effect was analyzed according to the thickness of the bottom MO TF. Figure 6 depicts J–V curves of the MO/Si HSCs with 8-, 15-, and 30-nm-thick MO TFs under dark and illuminated conditions. The solar cell parameters of the fabricated HSCs are outlined in Table 3. The highest PCE, 3.81%, was achieved when the 8-nm thick MO TF was used, and the PCE declined with increasing thickness of the MO TF. The drop in PCE is primarily attributed to increased R_s_ with 15- and 30-nm-thick MO TFs, 3.39 and 3.57, respectively, as shown in Table 3.

**Figure 7:**
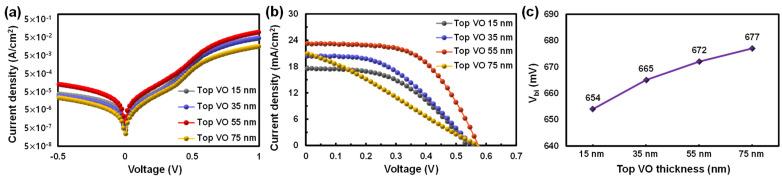
J–V curves of the TMT/Si HSCs according to the top VO layer thickness under (**a**) dark and (**b**) light (AM1.5) conditions. (**c**) V_bi_ values of the fabricated TMT/Si HSCs according to the top VO layer thickness.

Furthermore, the effect of the top VO thickness on TMT/Si HSC performance was investigated. To this end, the TMT/Si HSCs were fabricated with the bottom MO and middle Ag layer thicknesses fixed at 8 nm and 15 nm, respectively, and the top VO with varying thicknesses of 15 nm, 35 nm, 55 nm, and 75 nm was deposited. The changes in solar cell parameters and $\tau_{eff}$ according to the top layer thickness were analyzed. Figure 7 and Table 4 show the J–V curves and solar cell parameters under dark and illuminated conditions as a function of the thickness of the top VO, respectively. The values in Table 4 indicate that as the thickness of the TTMO increased, the PCE of the fabricated device increased. The highest PCE (7.57%) was achieved when the top VO has a thickness of 55 nm. However, the sample with 75 nm of top VO TF showed a lower PCE (3.33%). This drop in PCE is mainly attributed to the increased R_s_, 12.46 Ω${\mathrm{cm}}^{2}$, with 75 nm compared to 2.91 Ω${\mathrm{cm}}^{2}$ of 55 nm as shown in Table 4.

The improvement in PCE with the thicker top VO TFs is because the work function of the top VO becomes more dominant at the TMO/Si interface as the thickness increases [30,31]. However, in this TMT structure, the work function of top VO would be inevitably pinned at that of inserted metal (i.e., Ag) layer. Therefore, to expect thickness effect of top VO TF, the deposited Ag layer should not fully cover the MO surface to offer direct contact between top VO and bottom MO TFs over a certain fraction of the area. Therefore, before further discussion on the thickness effect of top VO TF, the morphology of the deposited Ag layer was measured with AFM, and the results are shown in Figure S1. For AFM, three different Ag thicknesses (i.e., 10, 15, 20 nm) were fabricated to investigate Ag thin film growth mechanisms in addition to their morphologies. From the AFM results, the deposited Ag layer on the MO surface produced discontinuous island-shaped surface morphologies for all three samples. which indicate that dominant thin-film growth mechanism for Ag layer on the MO surface is the Volmer–Weber growth mechanism. Based on these AFM results, it is clear that the top VO TF would form direct contact with the bottom MO TF over a certain area fraction to weaken the fermi-level pinning with Ag. (Note: in Figure S2 and Table S1, the measured J–V curve and the device parameters of TMT/Si HSCs are also provided, which showed that a 15 nm Ag layer is the optimal thickness for HSC fabrication.)

**Figure 8:**
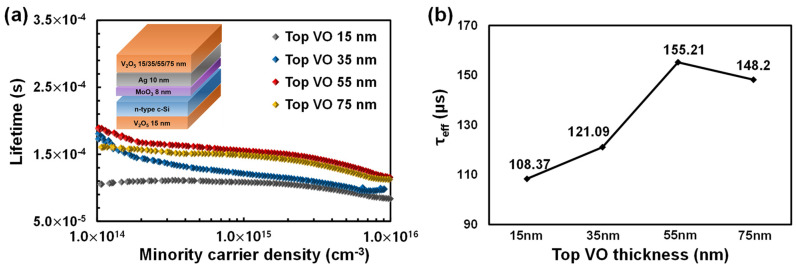
(**a**) Minority carrier lifetimes of the TMT multilayer deposited with various top VO thicknesses; (**b**) $\tau_{eff}$ values at the carrier injection level of 1.5 × 10^15^ cm^−3^.

The work function values for MO and VO are known to be similar at 6.9 and 7.0 eV, respectively [16,32]. However, as shown from the XPS results presented in Table 2, the stochiometric V_2_O_5_ ratio of VO is higher than the ratio of the oxidation state of MO; thus, its work function value is also predicted to be higher than that of MO. This is because the reduction of the oxygen vacancy density induces a decrease in the free carrier concentration inside the TMO, thereby weakening the n-type doping effect [33,34]. Therefore, with the increasing thickness of the top VO, VO work function value increases; a higher value is predicted to dominate the behavior at the TMO/Si interface. Consequently, as the thickness of the top VO increases, the built-in potential (*V_bi_*) of the TMO/Si interface increases, and this increase may lead to (1) reduced surface recombination loss because of the strengthened field-effect passivation effect; and (2) higher open circuit voltage (*V_oc_*) for the fabricated HSCs [35,36]. To validate the assumption, the current density–voltage diode equation (Equation (1)) was applied to the J–V curve (Figure 7a) obtained from the fabricated HSCs under the dark condition, and the saturation current (*J*_0_) was calculated from Equation (2):

(1) $$J=J_{0}\left(\exp\left(\frac{eV}{nkT}\right)-1\right);$$

(2) $$J_{0}=A^{*}AT^{2}\exp\left(-\frac{V_{bi}}{kT}\right),$$

where *A* is the contact area; A* is the effective Richardson constant (120 Acm^−2^K^−2^ for n-type silicon); *T* is 25 °C (298 K); *k* is the Boltzmann constant; *n* is the ideality factor; *J*_0_ is the reverse saturation current density; *V_bi_* is the barrier height in Schottky diodes (i.e., a built-in-potential); and *q* is the elementary charge. In this way, *V_bi_* was extracted and presented in Figure 7c [37]. In Figure 7c, it can be seen that the calculated *V_bi_* increased with the increasing top VO thickness. The increase in the *V_oc_* observed in Figure 7c reflects and confirms the influence of *V_bi_* on the solar cell parameters. Furthermore, the higher *V_bi_* induces an imbalance in electron and hole concentrations owing to the strengthened field-effect at the TMO/Si interface—this is expected to reduce the carrier loss caused by the Shockley–Read–Hall recombination (*R_SRH_*) at the Si surface as expressed in Equation (3); this, in turn, extends the $\tau_{eff}$ of the device [37,38].

(3) $$R_{SRH}=\left(n_{s}p_{s}-n_{i}{}^{2}\right)v_{th}\times \int_{E_{v}}^{E_{c}}\frac{D\left(E_{t}\right)}{(n_{s}+n_{1})/\sigma_{p}\left(E_{t}\right)+\left(p_{s}+p_{1}\right)/\sigma_{n}\left(E_{t}\right)}dE_{t},$$

where *n*_1_ and *p*_1_ are the densities of the electrons and holes in the bulk, respectively; n_s_ and *p_s_* are the densities of the electrons and holes at the surface, respectively; *v*th is the thermal velocity; *E_c_* and *E_v_* are the conduction and valance band energies, respectively; *D_it_* is the density of the interface states; and *σ_n_* and *σ_p_* are the energy-dependent capture cross sections of the holes and electrons, respectively. Figure 8 shows the measured $\tau_{eff}$ values as a function of the top VO thickness. The $\tau_{eff}$ value increased as the top VO thickness increased, as predicted. The $\tau_{eff}$ was recorded its highest value (155.2 μs) at a thickness of 55 nm, and it decreased somewhat at 75 nm but still remained high. Thus, for the fabrication of the TMT/Si HSC with different TMOs, increasing the TTMO thickness up to a value below the critical thickness (the thickness at which the device resistance increases) is expected to improve the overall PCE of the HSC.

### 3.3. Comparison between Asymmetric and Symmetric TMT/Si HSCs

**Figure 9:**
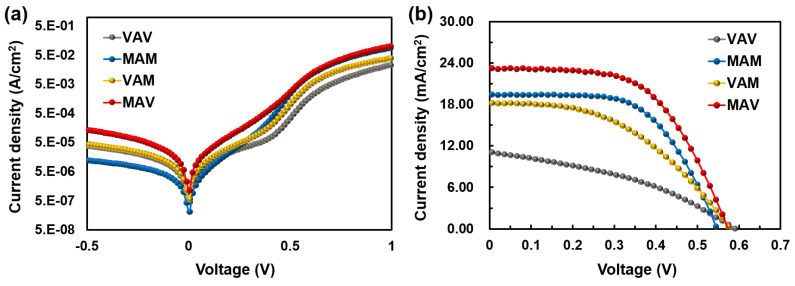
J–V curves of the fabricated TMT/Si HSCs under (**a**) dark and (**b**) light (AM1.5) conditions.

In this section, we compared the solar cell performance and long-term stability of the asymmetric TMT/Si HSC with the conventional symmetric TMT/Si HSC having one kind of TMOs for BTMO and TTMO. For the symmetric TMT/Si HSCs, samples for comparison were fabricated with structures of MO (8 nm)/Ag (15 nm)/MO (55 nm) (MAM) and VO (8 nm)/Ag (15 nm)/VO (55 nm) (VAV). For the asymmetric TMT/Si HSCs, samples were fabricated with structures of MO (8 nm)/Ag (15 nm)/VO (55 nm) (MAV) and VO (8 nm)/Ag (15 nm)/MO (55 nm) (VAM). Figure 9 shows the J–V curves of the fabricated HSCs under dark and illuminated conditions, and the solar cell parameters of each type of HSCs are presented in Table 5.

**Figure 10:**
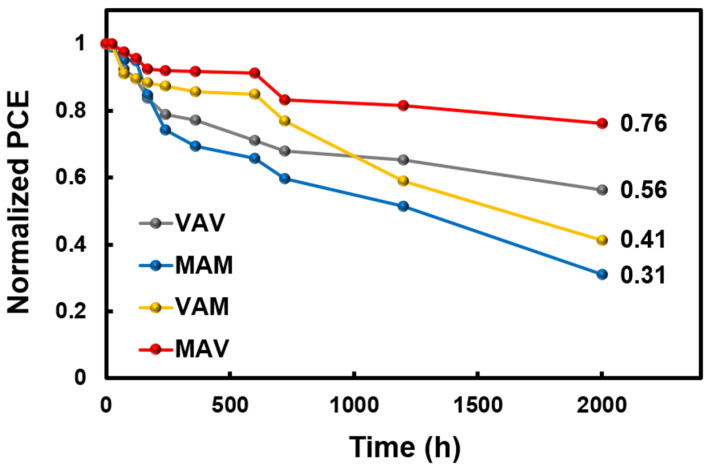
PCE degradation ratios of the fabricated TMT/Si HSCs over 2000 h.

Among the four different TMT/Si HSCs, the HSCs that applied MO as the BTMO, MAM and MAV, exhibited higher PCEs compared to those of VAV and VAM. This is attributed to an increase in the charge extraction efficiency of the photogenerated carrier, resulting from the low resistance of the MO layer itself. However, in the case of MAM (where MO was also applied as the TTMO), as shown in Figure 10, due to the formation of excessive oxygen vacancies in the MO layer, rapid PCE degradation over time was observed in relation to other HSCs used for comparison; this reveals the undesirable poor long-term stability of the HSC. However, in the case of the asymmetric TMT/Si HSC with the MAV structure, with the application of MO as the BTMO and VO as the TTMO, the highest PCE of 7.57% was achieved; enhanced long-term stability was also observed. From these results, in the future, a high-efficiency TMT/Si HSC with long-term stability can be developed through additional stoichiometry control and thickness optimization of the TMOs.

## 4. Conclusions

In this study, an asymmetric TMT (TMO–Metal–TMO) structure was introduced to overcome the limitations of existing TMO/Si HSCs. The asymmetric TMT structure was achieved by depositing MO with low resistance as the BTMO and VO with improved field-effect passivation as the TTMO. The results showed that the oxygen vacancy density plays a crucial role in determining the electrical properties of TMO TFs, the Si surface passivation effect, and the long-term stability of the HSC. The VO TF demonstrated better Si surface passivation and long-term stability, but its high resistance resulted in a low PCE compared to the MO TF. By combining the advantages of both MO and VO, the asymmetric TMT/Si HSC showed 20% improved PCE and long-term stability compared to the conventional symmetric TMT/Si HSC. This is because (1) lower resistance with bottom MO having higher density of oxygen vacancy; and (2) the enhanced field effect passivation and long-term stability with top VO TF, revealing lower density of oxygen vacancy. Therefore, the proposed asymmetric TMT/Si HSC could be a promising solution for low-cost and high-efficiency c-Si-based solar cells with improved Si surface passivation and long-term stability.

## Figures and Tables

**Table 1:** Solar cell performance parameters of TMO/Si HSCs under light (AM1.5) conditions.

Sample	J_sc_ (mA/cm^2^)	V_oc_ (mV)	FF (%)	R_sh_ (Ω·cm^2^)	R_s_ (Ω·cm^2^)	PCE (%)
VO_75 °C	14.09	618	37.81	14,102	5.65	3.29 ± 0.09
MO_RT	13.29	603	43.20	13,291	3.54	3.46 ± 0.11

**Table 2:** Integrated peak area ratio of vanadium and molybdenum oxidation states of each TMO TF.

Sample	Ratio of V Oxidation State (%)			Sample	Ratio of Mo Oxidation State (%)	
	V^5+^	V^4+^	V^3+^		Mo^6+^	Mo^5+^
VO (@ 75 °C)	88.3	10.5	1.2	MO (@ RT)	80.5	19.5

**Table 3:** Solar cell characteristic parameters of the MO/Si HSCs according to the MO layer thickness under light (AM1.5) conditions.

MO Thickness	J_sc_ (mA/cm^2^)	V_oc_ (mV)	FF (%)	R_sh_ (Ω·cm^2^)	R_s_ (Ω·cm^2^)	PCE (%)
8 nm	14.07	606	44.73	14,089	2.10	3.81 ± 0.05
15 nm	14.16	605	42.84	14,166	3.39	3.67 ± 0.06
30 nm	12.68	607	45.16	12,666	3.57	3.48 ± 0.09

**Table 4:** Solar cell characteristic parameters of the TMT/Si HSCs according to the top VO layer thickness under light (AM1.5) conditions.

MO/Ag/VO	J_sc_ (mA/cm^2^)	V_oc_ (mV)	FF (%)	R_sh_ (Ω·cm^2^)	R_s_ (Ω·cm^2^)	PCE (%)
8 nm/15 nm/15 nm	17.54	537	48.36	17,566	6.31	4.56 ± 0.17
8 nm/15 nm/35 nm	20.40	552	45.37	20,394	4.19	5.11 ± 0.14
8 nm/15 nm/55 nm	23.24	574	56.78	23,207	2.91	7.57 ± 0.07
8 nm/15 nm/75 nm	21.08	573	27.57	21,051	12.46	3.33 ± 0.06

**Table 5:** Solar cell characteristic parameters of the fabricated TMT/Si HSCs under light (AM 1.5) conditions.

Sample Structures	J_sc_ (mA/cm^2^)	V_oc_ (mV)	FF (%)	R_sh_ (Ω·cm^2^)	R_s_ (Ω·cm^2^)	PCE (%)
VO 8 nm/Ag 15 nm/VO 55 nm (VAV)	11.11	591	37.76	11,146	5.47	2.48 ± 0.05
MO 8 nm/Ag 15 nm/MO 55 nm (MAM)	19.42	547	59.22	19,411	2.54	6.29 ± 0.22
VO 8 nm/Ag 15 nm/MO 55 nm (VAM)	18.20	579	45.90	18,313	7.85	4.84 ± 0.18
MO 8 nm/Ag 15 nm/VO 55 nm (MAV)	23.24	574	56.78	23,207	2.91	7.57 ± 0.07

## Data Availability

Not applicable.

## Supplementary Materials

The following supporting information can be downloaded at: https://www.mdpi.com/article/10.3390/ma16165550/s1, Figure S1: AFM images of Ag layers deposited on MO surface: (a) 10 nm, (b) 15 nm, and (c) 20 nm; Figure S2: J–V curves of the TMT/Si HSCs according to the middle Ag layer thickness under (a) dark and (b) illumination (AM1.5) conditions; Table S1: Solar cell characteristic parameters of the TMT/Si HSCs according to the middle Ag layer thickness under illumination (AM1.5) conditions.
