# Supplementary material for: Asymmetric TMO–Metal–TMO Structure for Enhanced Efficiency and Long-Term Stability of Si-Based Heterojunction Solar Cells
Source: Materials (Basel). 2023 Aug 9;16(16):5550. doi: 10.3390/ma16165550 (PMC10456859; doi:10.3390/ma16165550)
Supplement: Supplementary file 1 [file materials-16-05550-s001.zip › materials-2515012-supplementary.pdf]

# Supporting Information

## Asymmetric TMO–Metal–TMO Structure for Enhanced Efficiency and Long-term Stability of Si-Based Heterojunction Solar Cells

**Yoon-Chae Jung [1], Young-Jin Yu [1], Yu-Kyung Kim [1], Jin Hee Lee [2], Jung Hwa Seo [3] and Jea-Young Choi [4,*]**

[1] Department of Metallurgical Engineering, Dong-A University, Busan 49315, Republic of Korea
[2] Department of Chemical Engineering (BK21 Four Graduate Program), Dong-A University, Busan 49315, Republic of Korea
[3] Department of Physics, University of Seoul, Seoul 02504, Republic of Korea
[4] Department of Materials Sciences & Engineering, Dong-A University, Busan 49315, Republic of Korea
* Correspondence: cjy4395@dau.ac.kr

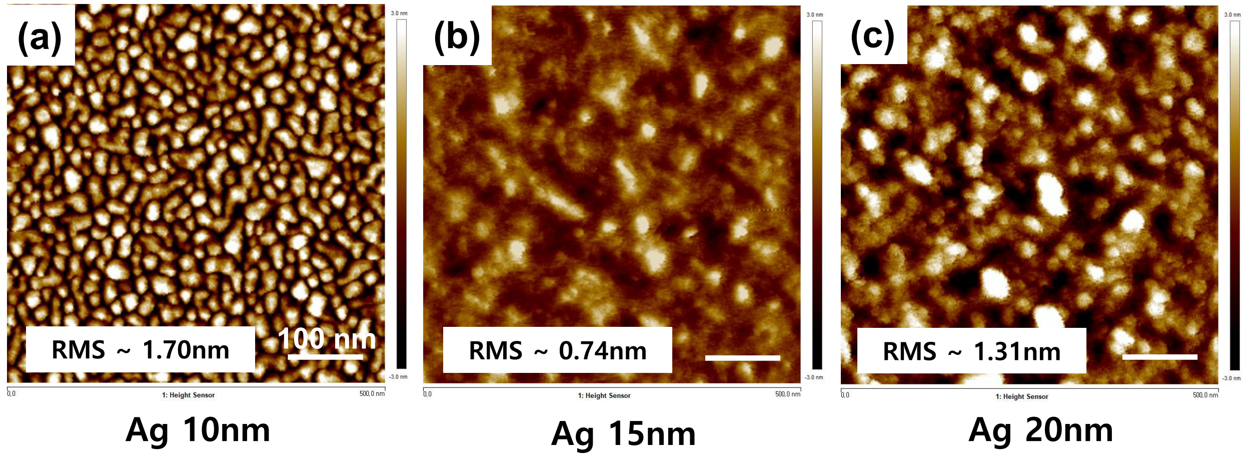


**Figure S1.** AFM images of Ag layers deposited on MO surface: (a) 10nm, (b) 15nm, and (c) 20nm.

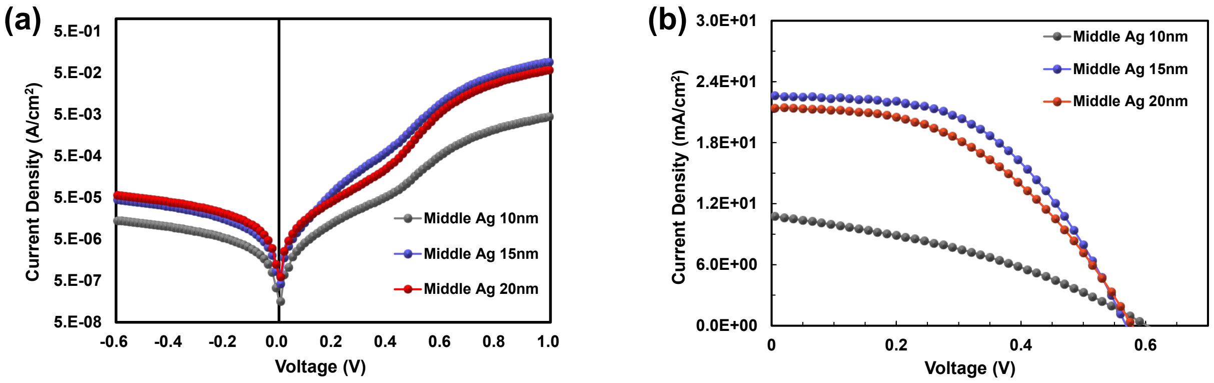


**Figure S2.** J–V curves of the TMT/Si HSCs according to the middle Ag layer thickness under (a) dark and (b) illumination (AM1.5) conditions.

**Table S1.** Solar cell characteristic parameters of the TMT/Si HSCs according to the middle Ag layer thickness under illumination (AM1.5) conditions.

| MO/Ag/VO | $J_{sc}$ ($mA/cm^2$) | $V_{oc}$ (mV) | FF (%) | $R_{sh}$ ($\Omega \cdot cm^2$) | $R_s$ ($\Omega \cdot cm^2$) | PCE (%) |
|---|---|---|---|---|---|---|
| 8 nm/10nm/55 nm | 10.79 | 603 | 36.10 | 10795 | 23.66 | 2.35 |
| 8 nm/15 nm/55 nm | 23.24 | 574 | 56.78 | 23207 | 2.91 | 7.57 |
| 8 nm/20 nm/55 nm | 21.44 | 579 | 46.04 | 21567 | 4.72 | 5.72 |
